# Estimation of Cardiometabolic Risk in Turkish Adolescents Using Different Anthropometric Techniques: Development and Temporal Validation of a Machine Learning Model

**DOI:** 10.3390/nu18142380

**Published:** 2026-07-21

**Authors:** Meryem Kahriman, Nihan Çakır Biçer, Murat Baş

**Affiliations:** 1 Department of Nutrition and Dietetics, Graduate School of Health Sciences, Acibadem Mehmet Ali Aydinlar University, 34752 Istanbul, Türkiye; nihancakir@gmail.com (N.Ç.B.); murat.bas@acibadem.edu.tr (M.B.); 2 Department of Molecular Gastroenterology and Hepatology, Institute of Gastroenterology and Hepatology, Kocaeli University, 41001 Kocaeli, Türkiye; 3 Department of Nutrition and Dietetics, Faculty of Health Sciences, Acibadem Mehmet Ali Aydinlar University, 34752 Istanbul, Türkiye

**Keywords:** cardiometabolic risk, triponderal mass index, body mass index, visceral adiposity index, lipid accumulation product index

## Abstract

Background: Although obesity is a recognized cardiometabolic risk determinant, debate persists over the most appropriate anthropometric technique. This study evaluated different anthropometric techniques for predicting cardiometabolic risk in Turkish adolescents and aimed to develop and temporally validate a machine learning–based prediction model, reported in accordance with the TRIPOD statement. Methods: Adolescents from the Türkiye Nutrition and Health Survey in 2010 (*n* = 1357) and 2017 (*n* = 561) were included. Anthropometric and biochemical parameters were assessed. BMI z-scores, waist-to-hip and waist-to-height ratios, triponderal mass index (TMI), visceral adiposity index (VAI), and lipid accumulation product (LAP) were calculated. Results: In ROC analysis, VAI showed the highest discrimination (AUC = 0.747, *p* < 0.001), followed by LAP (AUC = 0.670). In machine learning, XGBoost achieved the highest training discrimination (ROC–AUC = 0.879), whereas logistic regression was the most stable, with minimal overfitting (ΔAUC = 0.008). The logistic regression model was well calibrated (Brier score = 0.107; calibration slope 1.00 development, 1.03 external). External temporal validation showed comparable discrimination across models (AUC = 0.742–0.757), with logistic regression showing the greatest consistency. The model showed a high negative predictive value (NPV = 91.7%) and was therefore better suited to ruling out than ruling in cardiometabolic risk; positive findings warrant confirmatory testing (PPV = 35.4%). A simple logistic-based formula was derived. Conclusions: Machine learning approaches, together with the developed model and simplified formula, may be useful tools for estimating cardiometabolic risk in adolescents and could support a stepwise screening strategy, pending recalibration and prospective validation.

## 1. Introduction

Preventive programs have recently led to slight reductions in childhood obesity prevalence. However, it remains at an alarming level worldwide [1]. The World Health Organization [2] reported that in 2022, 160 million children and adolescents aged 5–19 years were obese. The Türkiye Nutrition and Health Survey (TNHS) 2017 data reported a 15% obesity prevalence among adolescents aged 15–18 years, drawing attention to the seriousness of the situation in our country [3]. Strong evidence exists that childhood obesity is a significant risk factor for cardiovascular and metabolic diseases [4,5]. In children and adolescents, cardiometabolic risk (CMR) is a metabolic condition characterized by the coexistence of abdominal obesity, dyslipidemia, glucose metabolism disorders, and high blood pressure [6,7,8]. Perinatal factors, age, sex, physical activity, nutritional status, and obesity/overweight are considered significant risk factors for this condition [6].

Strong evidence exists in the literature regarding risk factors, particularly obesity, showing that preventing and managing obesity is a crucial step in ruling out CMR in children [4,9]. However, how to screen for and identify obesity in children and adolescents remains debated. Worldwide, the body mass index (BMI) is broadly employed [10,11]. Moreover, age and sex-standardized BMI z-scores are widely used as a body composition indicator [12]. However, the effectiveness of BMI is debatable, especially for adolescents [10,13]. Based on this, Peterson et al. [10] defined the triponderal mass index (TMI), which is obtained by dividing body weight (kg) by cubic meters of height. Studies have highlighted that the TMI is at least as effective as BMI in determining obesity and body composition and predicting CMR [14,15,16,17]. Along with the TMI, abdominal obesity, expressed as simple ratios of anthropometric parameters (e.g., waist-to-hip and waist-to-height ratios) in adolescents, is also considered a simple and effective parameter in CMR prediction [18,19,20,21].

Alongside conventional anthropometric indices, new indices that encompass biochemical parameters have also been defined. One of these indices is the visceral adiposity index (VAI), which is calculated by incorporating waist circumference, BMI, triglycerides, and high-density lipoprotein cholesterol (HDL-C) into the formula [22]. The VAI represents a potential predictor of metabolic syndrome components, including glucose, triglycerides, HDL-C, and blood pressure in adolescents [22,23]. The lipid accumulation product (LAP) index, another index calculated based on waist circumference and triglyceride levels, can be another effective parameter in determining metabolic risk in adolescents [24]. Collectively, these findings underscore the potential of simple and practical anthropometric indices for predicting CMR in adolescents. However, evidence regarding their predictive performance in a nationally representative adolescent population in Türkiye remains scarce. Therefore, this study mainly aimed to evaluate the ability of anthropometric techniques to predict CMR among adolescents included in the TNHS datasets. Furthermore, our secondary aim was to develop and temporally validate a multivariable prediction model addressing this gap. The intended purpose of the model is risk stratification and stepwise screening (i.e., a clinical decision-support tool for ruling out low-risk adolescents and flagging those requiring confirmatory testing), rather than diagnosis; the study is reported following the TRIPOD reporting guideline for prediction model development and validation.

## 2. Materials and Methods

### 2.1. Study Design and Population

**Figure 1:**
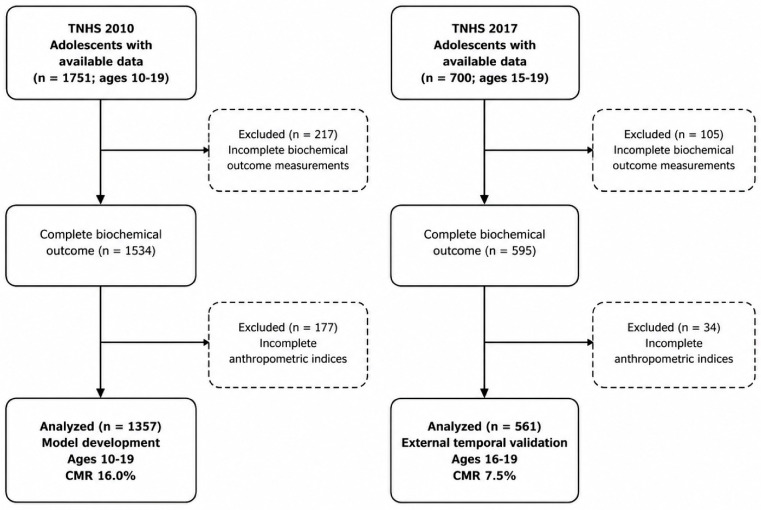
Flow of study participants. The TNHS 2010 dataset (ages 10–19) was used for model development and the TNHS 2017 dataset (ages 16–19) for external temporal validation. CMR, cardiometabolic risk (defined as ≥2 abnormal biochemical parameters). The visceral adiposity index and lipid accumulation product accounted for most of the missingness, as both require triglyceride and HDL-C values.

This cross-sectional study employed data from the TNHS conducted by the Ministry of Health between 2010 [25] and 2017 [3]. TNHS are cross-sectional field studies involving children/adolescents and adults, conducted using a multi-stage stratified cluster sampling method that is representative at the national level. Our study targeted the adolescent population aged 10–19 years as defined by the WHO [26]. In both datasets, individuals with complete biochemical parameters (glucose, total cholesterol, low-density lipoprotein cholesterol [LDL-C], HDL-C, and triglycerides) and anthropometric measurements (body weight, height, waist circumference, and hip circumference) were included in the analysis (Figure 1).

Participant flow was as follows. From the TNHS 2010 records, 1751 adolescents had available data; 217 were excluded for incomplete biochemical outcome measurements and a further 177 for incomplete anthropometric indices, yielding 1357 analyzed participants (aged 10–19 years) [27]. From the TNHS 2017 records, 700 adolescents had available data; 105 were excluded for incomplete outcome and 34 for incomplete anthropometric measurements, yielding 561 analyzed participants (aged 16–19 years) [3]. Missing biochemical values arose because not all participants had a complete fasting lipid and glucose panel; the visceral adiposity index and lipid accumulation product showed the highest missingness (17–18% in 2010; ~14% in 2017) because both require triglyceride and HDL-C values [3,27]. A complete-case approach was used, retaining only participants with complete outcome, anthropometric, and demographic data. All analyses were conducted at the individual participant level; survey sampling weights, clustering, and stratification were not applied, because the objective was to develop a portable individual-level prediction model rather than nationally representative prevalence estimates. Reported frequencies therefore describe the analytic sample rather than weighted population estimates. Included and excluded participants did not differ by sex in either survey wave (2010: 49.2% vs. 50.8% male, *p* = 0.63; 2017: 48.1% vs. 52.5% male, *p* = 0.41). Age did not differ between included and excluded participants in 2017 (17.7 vs. 17.7 years, *p* = 0.82); in 2010, excluded participants were slightly older than those included (14.9 vs. 14.0 years, *p* < 0.001). This small age difference is acknowledged as a potential source of selection bias in the Limitations.

### 2.2. Research Authorization and Ethical Approval

Before study initiation, institutional permission was obtained from the General Directorate of Public Health of the Ministry of Health of the Republic of Türkiye to use the TNHS 2010 and 2017 data. Furthermore, before applying for institutional permission, ethical committee approval (ATADEK-2025/02) was obtained from the Medical Research Evaluation Board of Acıbadem Mehmet Ali Aydınlar University. The TNHS 2010 and 2017 studies were conducted taking into account the principles of the Helsinki Declaration. Furthermore, written informed consent was obtained from all participants and/or their legal guardians prior to the start of the study.

### 2.3. Data Collection and Evaluation

In the TNHS 2010 and 2017, questionnaires containing questions about sociodemographic information, dietary habits, and general health status were administered to the participants. Anthropometric measurements were also taken, and blood samples were collected and analyzed. This study utilized the collected data.

### 2.4. Anthropometric Measurements

Participants’ body weights were measured while standing using a digital scale with a 0.1-kg sensitivity. Furthermore, their heights were measured while standing using a stadiometer with a 0.1-cm sensitivity. BMI was calculated as body weight (kg)/height (m)^2^ and evaluated using age- and sex-specific WHO growth reference z-scores (5–19 years) [28] via the WHO AnthroPlus program [29].

In addition, participants’ waist and hip circumference were measured using the technique recommended by the WHO [30]. Waist-to-hip and waist-to-height ratios were calculated on the basis of these data.

### 2.5. Biochemical Findings

Blood samples obtained from participants were analyzed in state hospitals or public health laboratories. TMI, VAI, and LAP indices were calculated as follows, using anthropometric measurements and biochemical findings:

**TMI:** Body weight (kg)/height (m^3^) [10].

**VAI:** For males: waist (cm)/(39.68 + 1.88 × BMI) × (TG [mmol/L]/1.03) × (1.31/HDL-C [mmol/L]).

For females: waist (cm)/(36.58 + 1.89 × BMI) × (TG [mmol/L]/0.81) × (1.52/HDL-C [mmol/L]) [31].

**LAP:** For males: (waist [cm] − 65) × TG (mmol/L).

For females: (waist [cm] − 58) × TG (mmol/L) [24].

### 2.6. Data Analysis

Continuous variables were summarized as means ± standard deviations (SDs), and categorical variables as frequencies and percentages. The performance of each anthropometric index in discriminating CMR was evaluated using receiver operating characteristic (ROC) curve analysis. The area under the curve (AUC) was calculated with a 95% confidence interval (CI), and the Youden index (J = Sensitivity + Specificity − 1) was employed for determining optimal cutoff points. Sensitivity, specificity, positive predictive value (PPV), negative predictive value (NPV), and likelihood ratios (LRs) were calculated for each cutoff point. The DeLong test was used for comparing AUC values of different anthropometric indices. Prediction models were developed using three different algorithms. Logistic regression modeling was applied as the primary multivariate analysis; model fit was evaluated using the LR test, Nagelkerke R^2^, and Hosmer–Lemeshow goodness-of-fit test. The random forest algorithm was implemented with 500 decision trees, class imbalance was corrected using downsampling, and hyperparameters (mtry, min.node.size, splitrule) were optimized using grid search. The XGBoost algorithm was trained using Bayesian optimization (20 initial models + 60 iterations), and L1 and L2 regularization was applied to avoid overfitting. All models were evaluated using 10-fold cross-validation and three iterations. Three repeats of 10-fold cross-validation (30 resamples) were used as a standard, computationally efficient scheme that yielded stable performance estimates with low variance across folds. For reproducibility, a fixed random seed (2025) was set for all resampling and model-fitting steps. Random forest hyperparameters were tuned over a grid of mtry (1–5), splitting rule (Gini, extratrees), and minimum node size (5, 10, 15, 20, 30, and 50), with 500 trees; XGBoost hyperparameters were tuned over the following ranges: number of trees 100–400, tree depth 2–4, minimum node size 10–30, mtry 2–4, learning rate 0.001–0.03, loss reduction (gamma) 0.1–10, subsample fraction 0.5–0.8, L2 regularization (lambda) 0.1–10, and L1 regularization (alpha) 0.01–1, using Bayesian optimization to limit model complexity. All hyperparameter tuning and class-imbalance correction were performed strictly within the cross-validation resampling folds to prevent information leakage, and optimal configurations are reported in Supplementary Tables S7 and S8. Package versions are provided in Supplementary Table S12. Model performance was evaluated using multiple metrics: ROC–AUC and area under the precision-recall curve (PR-AUC) for discrimination; Brier score for calibration; sensitivity, specificity, PPV, NPV, accuracy, balanced accuracy, F1-score, and Matthews correlation coefficient (MCC) for classification performance. The Youden index was used to determine optimal decision thresholds, and alternative threshold values were analyzed for different clinical scenarios (screening, balanced decision, and diagnostic confirmation). Model calibration was further assessed by the calibration slope and calibration intercept (calibration-in-the-large), together with flexible (loess) calibration curves, in both the development and external validation sets. The clinical utility of the final model was evaluated using decision curve analysis, quantifying net benefit across a range of threshold probabilities relative to the treat-all and treat-none strategies (Supplementary Figures S1 and S2). Models developed from TNHS 2010 data were applied to TNHS 2017 data to perform external temporal validation. During validation, the optimal threshold values determined in the 2010 training set were kept constant while simulating a real clinical application scenario. By calculating the internal–external AUC difference (generalization gap), the generalizability of the models was evaluated. Regarding the XGBoost model, SHapley Additive exPlanations (SHAP) analysis was applied to quantify the contribution of each variable to the model output. The mean absolute SHAP values were employed for identifying the variable importance ranking, dependency graphs were used for investigating the association and interactions between variable values and SHAP contributions, and waterfall plots were used for providing explanations of predictions at the individual level. The study was designed, conducted, and reported in accordance with the TRIPOD statement for the development and validation of multivariable prediction models; the completed checklist is provided in Supplementary Table S13. The sample size for model development was assessed against the criteria of Riley et al. using the pmsampsize package. For five candidate parameters, an outcome prevalence of 16.0%, and the observed performance (Cox–Snell R^2^ = 0.146; c-statistic = 0.757), the minimum required sample size was 282–370 participants (46–60 events). The available development sample (*n* = 1357; 217 events; 43 events per parameter) therefore substantially exceeded the minimum required to limit overfitting and ensure precise estimation. All statistical analyses were performed using the R 4.4.2 programming language. For data processing, tidyverse was used; for machine learning, caret and tidymodels; for Random Forest, ranger; for XGBoost, XGBoost; for ROC and precision–recall curve analysis, pROC and PRROC; for SHAP analysis, SHAPforxgboost and shapviz; and for graph generation, ggplot2 and patchwork packages were used [32,33,34,35,36,37,38,39,40].

Anthropometric-only indices (WHO BMI z-score, waist-to-hip ratio, waist-to-height ratio, and triponderal mass index) and indices incorporating biochemical parameters (VAI and LAP) were deliberately compared because they represent distinct screening paradigms: the former are non-invasive and suitable for community-level screening, whereas the latter require laboratory measurements and are more appropriate for a second, confirmatory tier. Evaluating both within the same population was a primary objective, allowing their relative performance and practical trade-offs to be quantified directly. The composite CMR score was defined using five biochemical parameters (glucose, total cholesterol, LDL-C, HDL-C, and triglycerides), with “high CMR” classified as the presence of at least two abnormal parameters. This threshold was selected because single-parameter abnormalities are common in adolescents [41], while the presence of multiple abnormalities reflects increased metabolic burden and supports the use of the CMR score as an early indicator of cardiometabolic impairment in this population (Supplementary Table S1). This ≥2 threshold was prespecified on the basis of the metabolic-clustering concept and prior literature, not derived by optimizing model performance. Blood pressure, fasting insulin, HbA1c, and inflammatory markers were not uniformly available across both survey waves and could therefore not be incorporated into the composite outcome; waist circumference was deliberately excluded from the outcome because it is used within the predictors (VAI, LAP, and waist-to-height ratio), which would otherwise introduce circularity. The outcome was thus restricted to the five biochemical parameters measured consistently in both waves. To assess whether the discrimination of the lipid-containing indices (VAI and LAP) was inflated by shared components with the outcome (incorporation bias), two sensitivity analyses were performed: first, the outcome was redefined using only the non-lipid parameters (glucose, total cholesterol, and LDL-C), and the single-predictor ROC analyses were repeated; second, VAI was removed from the final logistic regression model, and the resulting AUCs were compared with those of the full model using the DeLong test in both the development (2010) and external validation (2017) sets (Supplementary Table S11).

## 3. Results

### 3.1. Description of the Study Sample

Participants with suitable age ranges and available biochemical and anthropometric data were selected from the TNHS 2010 and 2017 datasets. Furthermore, after missing value analysis, analyses were conducted with a total of 1918 participants: 1357 from TNHS 2010 and 561 from TNHS 2017 for the external validation phase (Supplementary Table S2).

The mean age of the participants was 14.00 ± 2.66 years in TNHS 2010 and 17.68 ± 1.00 years in TNHS 2017. Furthermore, the mean WHO BMI z-score was 0.06 ± 1.25 in TNHS 2010 and 0.19 ± 1.28 in TNHS 2017 (Table 1).

When biochemical parameters were examined, the highest percentages outside the reference range in both periods were for LDL-C (26.5% of participants in TNHS 2010 and 20.1% in 2017) (Supplementary Table S1).

**Figure 2:**
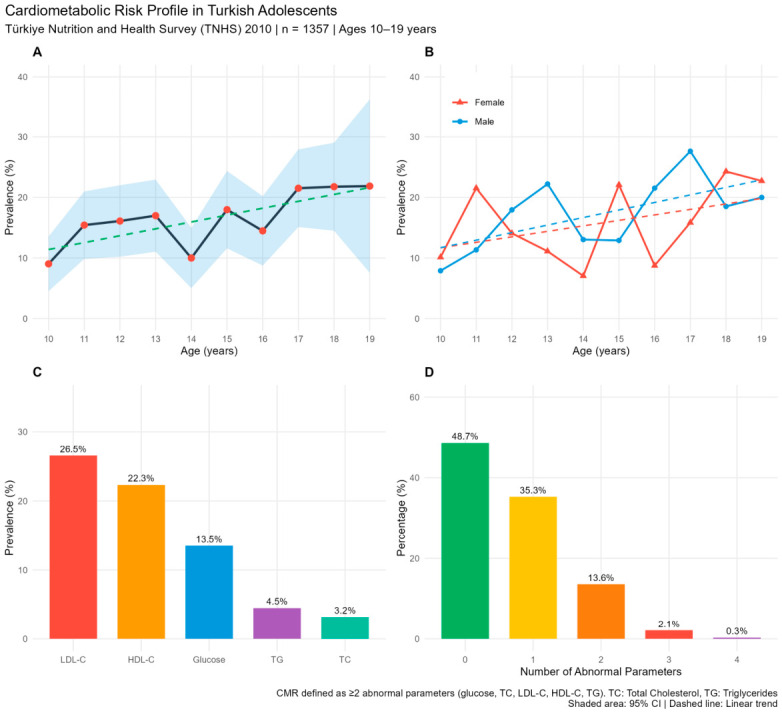
Cardiometabolic risk profile in Turkish adolescents. (**A**) Overall prevalence of cardiometabolic risk (CMR) by age. The solid black line represents the observed prevalence, the green dashed line indicates the linear trend, and the shaded area represents the 95% confidence interval. (**B**) Age-specific prevalence of CMR according to sex. Solid red and blue lines represent female and male prevalence, respectively, whereas the corresponding dashed lines indicate the linear trends. (**C**) Prevalence of individual cardiometabolic risk factor abnormalities (LDL-C, HDL-C, glucose, triglycerides, and total cholesterol). (**D**) Distribution of the number of abnormal cardiometabolic risk parameters among adolescents.

According to TNHS 2010 data, CMR was detected in 16.0% (*n* = 217/1357) of Turkish adolescents aged 10–19 years. The prevalence of CMR shows a significant increasing trend with age (Figure 2A,B). The most common abnormalities were in LDL-C (26.5%) and HDL-C (22.3%) levels (Figure 2C). 13.6% of adolescents had two parameter abnormalities and 2.4% had three or more parameter abnormalities (Figure 2D).

**Figure 3:**
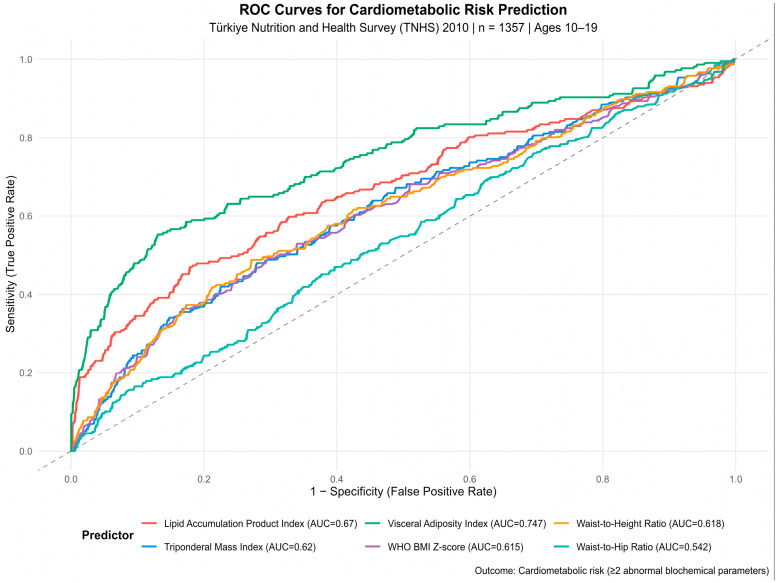
ROC curves for cardiometabolic risk prediction. The discriminative performance of six anthropometric indices in predicting cardiometabolic risk (≥2 abnormal biochemical parameters) is shown. The dashed line represents the reference line (AUC = 0.50).

Among the indices examined, VAI showed the highest discriminatory performance (AUC = 0.747; *p* < 0.001), while LAP showed the second highest (AUC = 0.670; *p* < 0.001). No statistically significant difference was observed between WHO BMI z-score (AUC = 0.615; J = 0.198), waist-to-height ratio (AUC = 0.618; J = 0.217), and TMI (AUC = 0.620; J = 0.200) (*p* > 0.05). Waist-to-hip ratio showed the lowest performance (AUC = 0.542; J = 0.075) and had limited diagnostic value (Figure 3, Supplementary Table S3).

VAI demonstrated the highest discriminative performance among the indices evaluated, particularly in predicting abnormal triglyceride (AUC = 0.976) and abnormal HDL-C levels (AUC = 0.763). Similarly, the LAP index exhibited a high AUC value for triglyceride abnormality (AUC = 0.839). No anthropometric index was successful in predicting abnormal glucose levels (all AUC values were 0.47–0.50).

### 3.2. Machine Learning

Before machine learning models were built, the basic assumptions of logistic regression were systematically evaluated. Logistic regression assumptions were met, including moderate multicollinearity (max VIF = 8.05), verified linearity (Box-Tidwell, *p* > 0.05), and sufficient model stability (EPP = 43.4). Therefore, the model was considered suitable as a basic comparator for machine learning models.

Only VAI was significantly associated with CMR (OR = 3.10; *p* < 0.001), indicating an approximately threefold increase in risk per unit increment. Other anthropometric indices were not statistically significant. The model demonstrated acceptable discrimination (AUC = 0.734 with cross-validation; 0.746 in the training set) and good calibration (Brier score = 0.107). The optimal threshold value determined using the Youden J index for CMR screening was 0.211, which provided 54.4% sensitivity and 87.6% specificity (Supplementary Table S4). Detailed classification performance metrics are shown in Supplementary Table S5, and the confusion matrix results are shown in Supplementary Table S6. Overall, the model performed well in excluding low-risk individuals but showed limited performance in positive-risk classification.

### 3.3. Random Forest and XGBoost Model Development Process

During random forest model development, hyperparameter tuning and class imbalance correction were applied. Despite perfect training performance (AUC = 1.00), the default model showed a marked decline in cross-validation (CV AUC = 0.715), indicating severe overfitting, particularly for the minority class. Among the rebalancing strategies, downsampling achieved the highest CV AUC (0.728) and the most balanced performance, whereas upsampling increased sensitivity at the expense of specificity and SMOTE provided intermediate results. However, none of the optimized RF models outperformed logistic regression. Variable importance analysis consistently identified VAI as the dominant predictor, followed by LAP, while other anthropometric indices contributed minimally, supporting the stability of the model outputs (Supplementary Table S7).

During XGBoost model development, hyperparameter tuning was performed using restricted ranges to limit model complexity, and class imbalance was addressed using downsampling, upsampling, and SMOTE. Although the upsampling model achieved the highest cross-validated AUC (0.735), its performance was comparable to logistic regression and did not represent a clinically meaningful improvement. Across all sampling strategies, XGBoost models exhibited high sensitivity (80–84%) but consistently lower specificity (52–58%). Optimal configurations favored low iteration numbers (nrounds = 50) and shallow trees, indicating low dataset complexity. Variable importance rankings were highly consistent, with VAI emerging as the dominant predictor (100%), followed by the LAP index, while other anthropometric indices contributed minimally and waist-to-hip ratio was negligible. Based on the Youden J index, no XGBoost model surpassed the sensitivity–specificity balance achieved by logistic regression (maximum J ≈ 0.375, logistic regression J = 0.420), indicating that although XGBoost increased sensitivity, logistic regression remained superior for CMR screening (Supplementary Table S8).

### 3.4. Final Model Configuration

Based on variable importance analyses, the model was simplified to improve stability. TMI showed no contribution in logistic regression, while waist-to-hip ratio consistently had zero importance in all machine learning models and was therefore excluded. Age was retained due to its relevance to metabolic risk, whereas sex was excluded because VAI and LAP are already sex-specific, potentially increasing redundancy. Predictors in the final model were retained on the basis of prespecified clinical relevance and their consistent contribution across the machine-learning models (variable importance), rather than by stepwise selection on statistical significance in the development sample; this approach avoids the optimism and unstable coefficient estimates associated with significance-based selection in prediction modeling. Consequently, some retained predictors (e.g., LAP, WHO BMI z-score, waist-to-height ratio) were not individually statistically significant but were kept to preserve model stability and clinical interpretability. The final model structure is presented in Table 2.

The wide confidence interval for the waist-to-height ratio coefficient (Table 2) reflects its small absolute measurement scale (producing a large per-unit coefficient) and its collinearity with other adiposity measures (maximum VIF = 8.05); this predictor was retained for clinical interpretability but its individual coefficient should be interpreted with caution. Model stability was evaluated by comparing training and cross-validated AUC values. Logistic regression showed the lowest overfitting (ΔAUC = 0.01), while random forest and XGBoost exhibited moderate (0.09) and substantial (0.15) overfitting, respectively. After strict regularization and Bayesian hyperparameter optimization, XGBoost’s overfitting was reduced (ΔAUC = 0.06), with improved generalization. Random forest required no further tuning and demonstrated acceptable CV performance (Supplementary Table S9).

Overall, although unregulated XGBoost achieved the highest training discrimination, logistic regression was the most stable model, and regularized XGBoost provided the best balance between discrimination and generalizability, with favorable specificity, calibration, and clinical utility. Final model selection should be guided by external validation (Supplementary Table S9).

### 3.5. Temporal External Validation

Temporal external validation was performed using TNHS 2017 data (*n* = 561; ages 16–19). All models demonstrated acceptable discrimination (ROC–AUC, 0.742–0.757), with logistic regression achieving the highest AUC (0.757) and minimal generalization gap. Random forest and regularized XGBoost showed comparable but slightly lower performance. No statistically significant differences in AUC were observed between models (DeLong test, *p* > 0.05). At fixed thresholds, random forest showed high sensitivity but very low specificity, whereas logistic regression and regularized XGBoost demonstrated more balanced performance. Threshold reoptimization increased specificity and PPV at the expense of sensitivity across all models (Table 3). Overall, the logistic regression model excelled with the highest generalizability, the lowest performance bias, and the advantage of interpretability. Calibration analysis confirmed these findings. In the development cohort, calibration was near-ideal (calibration slope = 1.00, intercept = 0.00; Brier score = 0.107). In the external cohort, the calibration slope remained close to unity (1.03), indicating that predictor effects were preserved over time, whereas the negative calibration intercept (−1.28) reflected systematic over-prediction driven by the lower outcome prevalence in 2017 (7.5% vs. 16.0%). This calibration-in-the-large offset can be corrected by simple intercept recalibration without refitting the model (Supplementary Figure S1). Decision curve analysis showed that, in the development cohort, the model provided a positive net benefit exceeding both the treat-all and treat-none strategies across the clinically relevant threshold range (approximately 0.10–0.30); in the external cohort, the absolute net benefit was smaller owing to the lower prevalence and calibration offset, while remaining at least as beneficial as the reference strategies at low thresholds (Supplementary Figure S2). The lack of statistically significant differences between the models (*p* > 0.05) supported the parsimony principle in the context of clinical decision support; accordingly, logistic regression was selected as the final model, whereas random forest and regularized XGBoost models were reported as analyses supporting sensitivity–specificity scenarios (Table 3).

**Figure 4:**
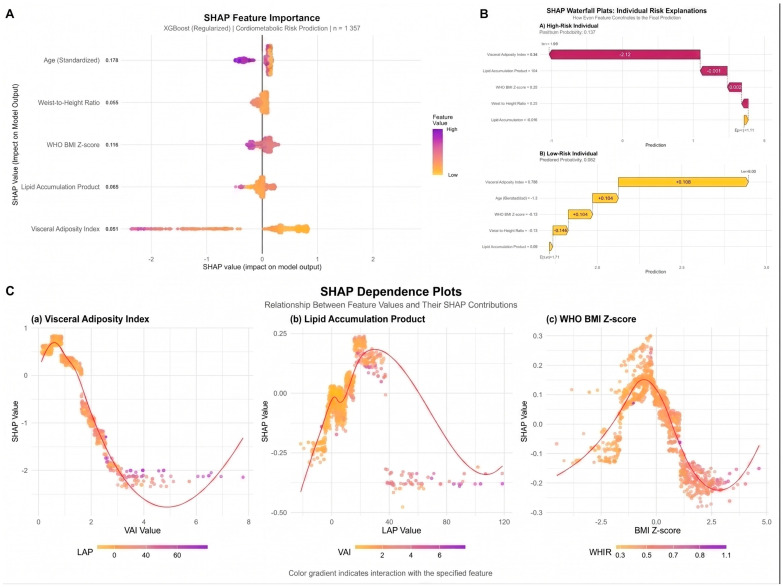
SHAP Analysis Figures for the XGBoost Model (**A**): SHAP feature significance plot showing the contribution of each variable to the XGBoost model. (**B**): SHAP waterfall plots showing individual prediction explanations for representative high- and low-risk individuals. (**C**): SHAP dependence plots showing the relationship between feature values and SHAP values. The red curves represent locally smoothed trend lines illustrating the relationship between feature values and SHAP values. In panel (**B**), the colored bars indicate the direction and magnitude of each feature’s contribution to the individual prediction. (VAI, Visceral adiposity index; LAP, lipid accumulation product index).

SHAP analysis was conducted only for the XGBoost model. Negative SHAP values indicated increased CMR probability. VAI was the dominant predictor (mean |SHAP| = 0.661), exceeding the combined contribution of all other variables (0.434). Age ranked second (26.9%), followed by WHO BMI Z-score (17.5%), LAP (12.8%), and waist-to-height ratio (8.3%) (Figure 4A). A clear threshold effect was observed for VAI at 1.63: SHAP values were positive below this level (≈0.62) and strongly negative above it, reaching −1.82 at VAI > 2.5 and stabilizing at −2.14 to −2.21 in the 3–5 range. WHO BMI Z-score also showed nonlinearity, with peak positive SHAP at Z ≈ −0.31 (≈0.30) and negative contributions above Z > 1. Individual explanations confirmed that extreme VAI values primarily drove risk predictions (Figure 4B). In individual-level analyses, a very high VAI (≈6.9) drove increased CMR risk (predicted probability ≈ 0.74), whereas a low VAI (≈0.8) exerted a protective effect (predicted probability ≈ 0.05), with other variables contributing modestly (Figure 4C).

**Figure 5:**
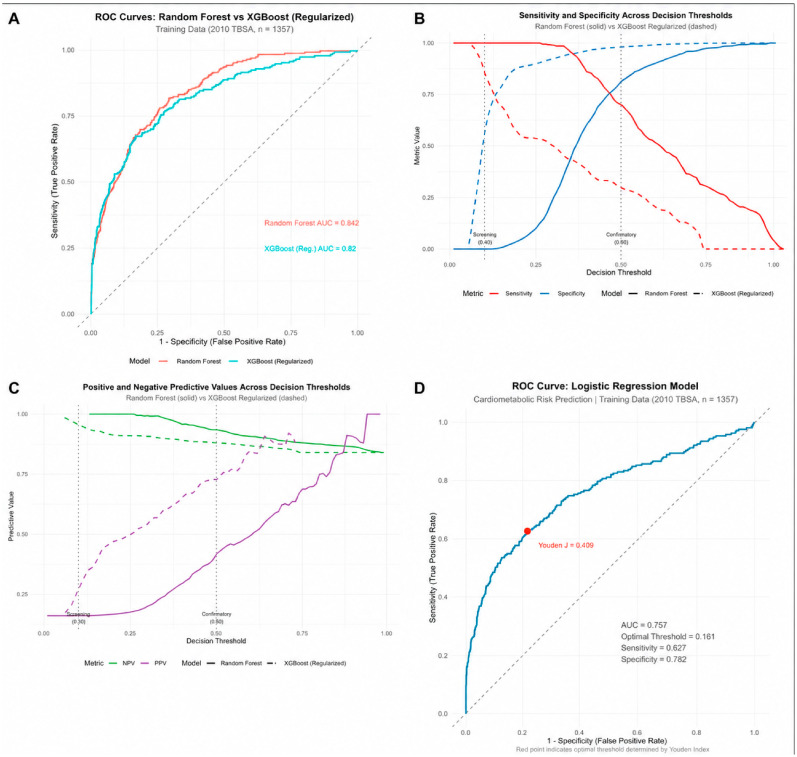
Clinical Decision Thresholds and Scenario Analysis Figures (**A**): ROC curves of the random forest and XGBoost (regularized) models; the grey dashed diagonal represents the line of no discrimination. (**B**): Changes in sensitivity (red) and specificity (blue) according to decision thresholds. Solid lines represent the Random Forest model, and dashed lines represent the XGBoost (regularized) model. Vertical grey dashed lines indicate the proposed screening (0.10) and confirmatory (0.50) decision thresholds. (**C**): Positive (PPV, purple) and negative (NPV, green) predictive values according to decision thresholds. Solid lines represent the Random Forest model and dashed lines represent the XGBoost (regularized) model. Vertical grey dashed lines indicate the proposed screening (0.10) and confirmatory (0.50) decision thresholds. (**D**): ROC curve and optimal threshold of the logistic regression model. The grey dashed diagonal represents the line of no discrimination, and the red point indicates the optimal threshold determined by the Youden Index. (NPV, negative predictive value; PPV, positive predictive value).

ROC analysis showed that both random forest (AUC = 0.842) and regularized XGBoost (AUC = 0.820) discriminated CMR across a wide range of thresholds, indicating adequate classification ability but also the need for threshold-specific evaluation for clinical decision-making (Figure 5A). Increasing the decision threshold reduces sensitivity and increases specificity. At low thresholds (<0.25), the random forest model provides 97–100% sensitivity, while specificity remains below 25%. When the threshold is increased to 0.50, sensitivity drops to the 30–50% range. A low threshold for screening purposes (0.10) and a high threshold for diagnostic confirmation purposes (0.50) correspond to different clinical scenarios (Figure 5B). As shown in Figure 5, in low-prevalence populations (16%), despite high negative predictive value (99–100%), positive predictive value is limited at low thresholds (16–20%); increasing thresholds increase PPV at the expense of a slight decrease in NPV; this indicates that in low-prevalence populations, negative results reliably rule out risk, while positive results require confirmation (Figure 5C).

Two clinical scenarios were defined to address different needs. For community-based screening, prioritizing sensitivity, the random forest model achieved 99.1% sensitivity and 99.3% NPV at a 0.30 threshold, identifying 215 of 217 at-risk adolescents at the cost of a high false-positive rate (75.5%), an acceptable trade-off for screening. For diagnostic confirmation, prioritizing specificity and PPV, the XGBoost model achieved 97.9% specificity and 72.7% PPV at a 0.50 threshold (LR+ = 14.01), indicating strong risk enrichment among test-positive individuals (Supplementary Table S10; Supplementary Figure S2). Across models, no significant performance differences were observed (DeLong test, all *p* > 0.05); however, logistic regression showed the greatest CV–external consistency with minimal generalization gap (0.008), supporting its robustness across populations (Supplementary Table S10).

The ROC analysis showed that logistic regression achieved good discrimination (AUC = 0.757, 95% CI: 0.717–0.796), with an optimal Youden-based threshold of 0.161 yielding moderate sensitivity (62.7%) and specificity (78.3%), high NPV (91.7%) for excluding CMR, and a relatively low PPV (35.4%) consistent with the low-prevalence setting (Figure 5D).

According to logistic regression, the probability of CMR is formulated as follows:

$$logit(P)=\;-2.942\;+\;1.193\times VAI\;+0.195\times {Age}_{std}+\;0.151\times BMI_{Z}\;-0.004\times LAP\;-\;0.880\times WHtR$$

## 4. Discussion

CMRs remain a public health concern among adolescents, with long-term consequences for individuals and society [42,43]. Although obesity is considered a significant factor for CMR in this population [9], the ideal anthropometric technique for screening remains unclear [10,13]. Accordingly, we evaluated the effectiveness of anthropometric parameters in predicting CMR among adolescents participating in the nationally representative TNHS. Furthermore, machine learning-based analyses were conducted.

In TNHS 2010, which we used as the training data in machine learning, the CMR (≥2 abnormal biochemical parameters) in adolescents was 16%. A national study conducted in Korea reported that the prevalence of ≥2 CMR factors among adolescents with obesity was 49.8% [4]. A study conducted in Türkiye on children with overweight/obesity aged 5–19 years reported that the prevalence of metabolic syndrome was 22%, and the prevalence of CMR was 46.6% [44]. These findings indicate that the CMR profile can vary depending on the population. Moreover, we observed that CMR tends to increase with age. This finding supports that of a previous study in Türkiye on children and adolescents with obesity, showing that metabolic syndrome is more common in older children [45].

When evaluating anthropometric indices in predicting CMR, we observed the highest discriminative performance and AUC in VAI. Vizzuso et al. [46], in their study evaluating the relationship between VAI and metabolic syndrome, revealed that VAI is associated with HOMA-IR, systolic blood pressure, LDL, HDL, triglycerides, and triglyceride-HDL ratio, and is an important parameter in predicting metabolic syndrome. Another study showed that VAI performed well in predicting CMR in adolescents with altered blood pressure and insulin resistance [47]. All these findings confirm that VAI is promising as a predictor of CMR. Furthermore, our study revealed that VAI exhibited high discriminative performance in predicting abnormal triglyceride levels, which is partly expected given that triglycerides are a component of the VAI formula; this can be attributed to the inclusion of triglyceride levels in the VAI formulation [48]. Moreover, the LAP index showed the second best performance in predicting CMR. Comparably with our findings, Tamini et al. [49] confirmed that LAP performs well in diagnosing metabolic syndrome and is a diagnostic tool that can be used in clinical practice. Regarding traditional anthropometric techniques, including BMI z-score, TMI, and waist-to-height ratio, we observed similar and moderate significance. Among these techniques, TMI in particular is accepted in the literature as a superior parameter to BMI in predicting obesity and CMR in adolescents [14,17,50,51]. However, our findings did not support this view and indicate the need for more comprehensive studies in this population. Furthermore, the diagnostic value of waist-to-hip and waist-to-height ratios [18,19,20,21], which are accepted techniques for predicting CMR in this age group, was found to be limited in our study.

Before proceeding to machine learning models, we systematically evaluated the basic assumptions of logistic regression. In the regression model, we noted that only the VAI was statistically significant. The high correlations observed between the WHO BMI z-score, waist-to-height ratio, and LAP index confirm that these measures largely reflect the same body composition components [49,52]. Notably, because VAI and LAP incorporate triglycerides and HDL-C, which are also components of the composite outcome, part of their discrimination reflects mathematical coupling (incorporation bias). Sensitivity analyses confirmed this: when the outcome was restricted to non-lipid parameters, VAI’s discrimination attenuated markedly (AUC 0.747 for the original outcome vs. approximately 0.57–0.62 for the non-lipid outcome) and was no longer the top-performing index, and removing VAI from the model reduced the AUC significantly in the development set (0.757 to 0.658; DeLong *p* < 0.001) though not in the smaller external set (0.757 to 0.683; *p* = 0.10) (Supplementary Table S11). VAI is therefore best interpreted as an efficient summary of lipid-related adiposity dysfunction rather than as an independent biomarker of cardiometabolic risk. To capture nonlinear associations and determine interactions between variables, we also evaluated the random forest and XGBoost models and compared them with logistic regression. The final predictor set was defined on the basis of prespecified clinical relevance and consistent contribution across model families (variable importance), rather than by significance-based stepwise selection, an approach that reduces optimism and coefficient instability in prediction modeling [53]. In this study, the fact that the TMI consistently exhibited near-zero significance in logistic regression and the waist-to-hip ratio consistently showed near-zero significance in the random forest and XGBoost models suggest that these variables do not offer additional information in distinguishing CMR, contrary to the literature [14,21,50,51]. The inclusion of age as a variable in the model agrees with the literature, showing that age influences the metabolic risk profile in adolescents [54]. In the comparisons, we observed that logistic regression was the most stable model, whereas random forest and especially XGBoost were more prone to overfitting when unadjusted. Importantly, although machine-learning methods were central to our analytical approach, they did not outperform conventional logistic regression: logistic regression showed the highest external-validation AUC, the smallest generalization gap, and the greatest stability, with no statistically significant AUC differences between models (DeLong *p* > 0.05). Our findings therefore indicate that, for this task and dataset, conventional regression remains highly competitive with more complex algorithms, consistent with the parsimony principle for clinical decision support. In this context, although the adjusted XGBoost model provides a balanced profile, the approach that the final decision should be made on the basis of temporal external validation results aligns with the modern predictive modeling literature [55]. The significant difference in CMR prevalence (16.0% vs. 7.5%) and age range between the training (2010) and external validation (2017) sets enabled testing model performance under varying conditions. The marked differences in age structure and outcome prevalence provide a more heterogeneous test of temporal transportability, but they also limit direct comparability between the development and validation cohorts. The lower cardiometabolic risk prevalence in 2017 (7.5% vs. 16.0%) cannot be attributed straightforwardly to the older age distribution, because cardiometabolic risk increased with age within the development cohort. It more likely reflects a combination of survey-wave sampling, secular changes, differences in laboratory completeness and selection, and other unmeasured cohort differences. The narrower age range in 2017 altered the case mix and may have affected transportability, but does not explain the direction of the prevalence difference. Because age distribution and prevalence differed, the temporal validation should be interpreted as a test of transportability across time and age structure rather than as replication under identical conditions. Predictive models will be applied in practice at different times, with different age distributions, and risk prevalences [55]. The consistency between external validation performance and internal validation results supports preservation of discrimination in this temporal transportability setting [56]. Nonetheless, because both datasets originate from the same national survey system and represent the same ethnic population, this constitutes temporal rather than fully independent external validation. Its geographic and ethnic transportability therefore remains untested, and performance may differ in populations with different obesity phenotypes, dietary patterns, and cardiometabolic risk distributions. Validation in ethnically diverse, non-Turkish adolescent cohorts is an important direction for future work.

Direct comparison with prediction models developed in other regions is informative but limited. In North America, a machine-learning model for adolescent metabolic syndrome developed from the National Health and Nutrition Examination Survey (NHANES, 2007–2016), using non-invasive predictors and interpreted with SHAP, reported very high apparent discrimination (best-model AUC up to 0.97) [57]. In Asia, a machine-learning model for pediatric metabolic syndrome derived from the Korean National Health and Nutrition Examination Survey and externally validated in an independent cohort combined anthropometric and bioelectrical-impedance parameters with SHAP-based interpretation [58]. In Europe and internationally, waist-to-height-ratio cut-offs for predicting cardiometabolic risk in children and adolescents aged 6–18 years have been derived and externally validated across multiple countries [59]. Our discrimination (AUC 0.757) is more modest than the highest of these reports; however, direct numerical comparison is constrained by substantial heterogeneity in the definition of the cardiometabolic outcome, in the predictor sets (several models incorporate blood pressure or the same lipid measures that also define the outcome, which can inflate apparent performance), in the validation design, and in the age range studied. Within this context, our results are consistent with the achievable range for anthropometry-based pediatric cardiometabolic risk prediction, and our incorporation-bias-aware, parsimonious modeling provides a conservative and transportable estimate.

The SHAP plots generated for VAI and WHO BMI Z-score reveal that the relationship between CMR and anthropometric indicators is nonlinear and includes significant threshold effects. These patterns are consistent with the VAI–CMR associations reported in the literature [46,47]; however, because SHAP values explain model predictions rather than biological processes, they should be interpreted as model-level, nonlinear heterogeneity that may be compatible with growth- and puberty-related differences during adolescence, not as evidence of causal biological mechanisms [60]. Similarly, the nonlinear relationship of BMI z-scores shows that growth and pubertal processes in adolescents complicate the relationship with CMR [61].

From a nutritional and metabolic perspective, the dominance of VAI is biologically coherent. VAI integrates waist circumference, BMI, triglycerides, and HDL-C and thus captures visceral and ectopic fat accumulation, the adipose depot most strongly linked to cardiometabolic dysfunction. Expansion of visceral adipose tissue promotes a pro-inflammatory, insulin-resistant state characterized by increased free fatty acid flux to the liver, hepatic triglyceride accumulation, atherogenic dyslipidemia (elevated triglycerides and reduced HDL-C), and impaired glucose handling. During adolescence, these processes are modulated by the nutritional transition toward energy-dense, high-saturated-fat and high-sugar diets and by pubertal changes in sex hormones and body composition, which transiently alter insulin sensitivity and fat distribution. This mechanistic background helps explain why an index reflecting lipid-related adiposity (VAI) discriminated cardiometabolic risk more effectively than indices of general adiposity (BMI z-score, TMI) in this population.

When individual SHAP explanations are evaluated, the strong and dominant contribution of VAI in individuals with the highest probability of prediction reveals that the model gives high weight to this variable, while the positive but limited contribution of VAI in low-risk individuals shows that the risk stratification is based on the interaction of multiple variables. This finding confirms that the SHAP method can explain the variable contributions in complex machine learning models at both global and individual levels, thus significantly minimizing the “black box” criticism [62]. It should be emphasized, however, that SHAP values and variable-importance rankings quantify each predictor’s contribution to the model’s output, not its biological or causal importance; they explain how the model reaches its predictions rather than the underlying pathophysiology. These results should therefore be interpreted as associational and model-specific rather than as evidence of causal effects. The model outputs are thus suitable for classification and risk profile interpretation.

ROC-AUC alone is insufficient for clinical decision-making; the decision threshold must be clearly defined and chosen in a manner consistent with the clinical context [63]. Threshold selection strongly influences predictive values, especially in low-prevalence populations. In this context, maintaining a high NPV at low thresholds may be useful for rule-out-oriented screening, which is a primary aim of screening tests. Conversely, a low PPV indicates that positive results should be confirmed using a second-stage test [64,65]. Accordingly, the two separate scenarios described in this study—a low threshold for community-based screening and a high threshold for diagnostic confirmation—are consistent with the risk-based decision approach suggested in the literature. In the present study, the reporting of different threshold values demonstrates that the model is not reduced to a single decision point, but is adaptable to clinical use cases. In practical terms, the combination of high NPV and modest PPV means that a negative result substantially lowers the likelihood of cardiometabolic risk and may help reduce unnecessary second-stage testing, whereas a positive result substantially enriches for risk but still includes many false positives and therefore warrants fuller cardiometabolic assessment rather than being treated as diagnostic. Because computing VAI already requires triglyceride and HDL-C measurement, the model is best positioned not as a pre-laboratory screen but as a second-tier tool that follows simple, non-invasive anthropometric triage and combines anthropometric and lipid information to flag adolescents who merit fuller clinical evaluation; it is thus used primarily as a rule-out tool. Decision curve analysis was consistent with this interpretation, showing a positive net benefit over the treat-all and treat-none strategies across screening-relevant thresholds in the development cohort (Supplementary Figure S2).

The derived risk prediction formula (logit(*P*) = −2.942 + 1.193 × *VAI* + 0.195 × ${Age}_{std}$+ 0.151 × $BMI_{Z}$ − 0.004 × *LAP* − 0.880 × *WHtR*) provides a transparent candidate risk-estimation tool that integrates multiple anthropometric and lipid-based indicators into a single probabilistic estimate; it is intended for independent evaluation and recalibration in future prospective cohorts rather than for immediate clinical use. An important practical caveat is that the strongest predictor, VAI, requires laboratory measurement of triglycerides and HDL-C, so the formula is not purely anthropometric and is therefore less suited to initial, community-level screening than fully non-invasive indices such as the waist-to-height ratio or TMI. This reinforces a stepwise approach: simple anthropometric measures can serve as a first, non-invasive triage step, with the laboratory-based model reserved for a second tier to refine risk estimates among those flagged.

This study has several limitations. Its cross-sectional design precludes causal inference, and the findings are limited to Turkish adolescents, restricting generalizability. The definition of CMR (≥2 abnormal biochemical parameters) involved subjective thresholding, and because VAI and LAP embed triglycerides and HDL-C, which also contribute to the outcome, their discrimination is partly affected by incorporation bias, as quantified in our sensitivity analyses (Supplementary Table S11). Because incorporation bias may inflate not only apparent discrimination but also the predictive values, including the negative predictive value on which the rule-out use depends, the observed performance should be interpreted cautiously until the model is validated against an outcome that is independent of the lipid components embedded in VAI and LAP. We also note that, because VAI incorporates both triglycerides and HDL-C, abnormalities in these two components alone can contribute substantially toward the ≥2-of-5 outcome definition. Key confounders such as physical activity, diet quality, socioeconomic status, and family history were not included. Predictors were intentionally restricted to anthropometric indices (plus age), consistent with the study’s aim of comparing anthropometric techniques; established determinants such as diet, physical activity, socioeconomic status, family history, pubertal status, and smoking were outside this scope and were not uniformly available across waves, which may limit overall performance and generalizability. The outcome also omitted blood pressure, insulin resistance, HbA1c, and inflammatory markers, restricting it to a lipid- and glucose-based construct. Analyses did not incorporate the complex survey design (sampling weights, clustering, and stratification); therefore, reported prevalences reflect the analytic sample rather than weighted national estimates, and the absence of survey-design adjustment may also affect coefficient estimates and their transportability. In addition, the complete-case approach excluded participants with missing measurements (approximately 20–23% of available adolescents, predominantly due to incomplete laboratory or anthropometric records), which could introduce selection bias if missingness was informative. Finally, the differing age structure of the training and validation datasets is a limitation; although it provided a heterogeneous test of temporal transportability, it also limited direct comparability between the cohorts. Moreover, although discrimination was preserved on external validation, the model systematically over-predicted risk in the 2017 cohort because of its lower outcome prevalence; before deployment in a new population, the model should therefore be recalibrated (e.g., by updating the intercept) and, ideally, prospectively validated.

## 5. Conclusions

Among the indices evaluated, VAI showed the highest discrimination for cardiometabolic risk in Turkish adolescents, although our sensitivity analyses indicate that part of this advantage reflects its shared lipid components rather than independent predictive value. The derived logistic regression formula integrates anthropometric and lipid-based information into a single estimate that showed a high negative predictive value, suggesting greater suitability for rule-out-oriented risk stratification than for rule-in decisions; positive results warrant a fuller cardiometabolic assessment rather than being interpreted diagnostically. Because VAI depends on laboratory measurements, the model is best positioned as a second-tier tool following simple, non-invasive anthropometric screening. Before clinical use, the model should be recalibrated for the target population and validated prospectively in diverse, ethnically distinct cohorts, and its cost-effectiveness assessed.

## Figures and Tables

**Table 1:** Anthropometric measurements, biochemical parameters, and indices by sex.

Variable	TNHS 2010							TNHS 2017						
	Males		Females		Total			Males		Females		Total		
	*n*	$\bar{x}$ ± SD	*n*	$\bar{x}$ ± SD	*n*	μ ± σ	Min–Max	*n*	$\bar{x}$ ± SD	*n*	$\bar{x}$ ± SD	*n*	μ ± σ	Min–Max
Age (year)	668	13.78 ± 2.62	689	14.21 ± 2.69	1357	14.00 ± 2.66	10–19	270	17.67 ± 1.01	291	17.68 ± 0.98	561	17.68 ± 1.00	16–19
WHO BMI z-score	668	0.04 ± 1.27	689	0.08 ± 1.22	1357	0.06 ± 1.25	−4.52–4.64	270	0.13 ± 1.37	291	0.25 ± 1.20	561	0.19 ± 1.28	−5.25–5.74
Waist-to-hip ratio	668	0.83 ± 0.07	689	0.78 ± 0.08	1357	0.80 ± 0.08	0.06–1.74	270	0.83 ± 0.07	291	0.78 ± 0.07	561	0.80 ± 0.07	0.52–1.25
Waist-to-height ratio	668	0.45 ± 0.06	689	0.45 ± 0.07	1357	0.45 ± 0.06	0.27–1.10	270	0.46 ± 0.06	291	0.47 ± 0.07	561	0.46 ± 0.06	0.34–0.77
Triponderal mass index	668	12.77 ± 7.01	689	13.28 ± 2.82	1357	13.03 ± 5.32	7.38–184.62	270	13.11 ± 2.66	291	14.12 ± 2.91	561	13.63 ± 2.84	7.57–32.43
Lipid accumulation product index	668	6.99 ± 15.43	689	10.51 ± 14.17	1357	8.78 ± 14.91	−21.53–118.69	270	18.08 ± 21.23	291	16.53 ± 14.60	561	17.27 ± 18.10	−5.93–128.03
Visceral adiposity index	668	1.00 ± 0.76	689	1.40 ± 0.93	1357	1.20 ± 0.87	0.11–7.78	270	1.20 ± 0.78	291	1.29 ± 0.71	561	1.25 ± 0.75	0.20–5.87
Glucose (mg/dL)	666	81.50 ± 13.10	686	79.19 ± 9.41	1352	80.33 ± 11.43	38.00–272.00	270	87.17 ± 10.00	291	85.13 ± 9.19	561	86.11 ± 9.63	37–131
Total cholesterol (mg/dL)	667	141.40 ± 29.14	687	146.53 ± 27.44	1354	144.00 ± 28.40	63.00–291.00	270	141.05 ± 25.46	290	153.07 ± 28.53	560	147.28 ± 27.73	78–305
LDL-C (mg/dL)	666	84.95 ± 23.48	687	87.93 ± 23.29	1353	86.46 ± 23.42	15.40–242.10	264	77.27 ± 21.74	287	83.67 ± 24.86	551	80.60 ± 23.61	18–227.4
HDL-C (mg/dL)	668	41.79 ± 10.67	689	43.98 ± 11.26	1357	42.90 ± 11.02	15.30–130.00	270	45.66 ± 10.98	291	53.46 ± 11.11	561	49.71 ± 11.71	26.6–117.8
Triglyceride (mg/dL)	668	73.09 ± 42.44	689	74.02 ± 36.20	1357	73.56 ± 39.38	12.00–385.00	270	92.33 ± 45.75	291	83.44 ± 33.43	561	87.72 ± 40.05	26–329

**Table 2:** Final base model logistic regression coefficients.

Variable	β	SE	OR	%95 CI	*p*
Constant	−2.942	1.028	0.053	0.007–0.409	0.004
VAI	1.193	0.129	3.297	2.573–4.269	<0.001
LAP	−0.004	0.011	0.996	0.975–1.018	0.706
WHO BMI z-score	0.151	0.099	1.164	0.958–1.415	0.127
Waist-to-height ratio	−0.880	2.300	0.415	0.004–31.669	0.702
Age (standardized)	0.195	0.092	1.216	1.016–1.456	0.033

**Table 3:** External temporal validation results (2017 test set, *n* = 561).

Metric	Logistic Regression	Random Forest	XGBoost (Reg.)
Discrimination			
ROC–AUC	0.757	0.742	0.744
%95 CI	0.664–0.850	0.649–0.834	0.644–0.844
Fixed Threshold (Training)			
Threshold value	0.161	0.439	0.164
Sensitivity	0.714	0.857	0.714
Specificity	0.622	0.256	0.622
PPV	0.133	0.085	0.133
NPV	0.964	0.957	0.964
Youden J	0.337	0.113	0.337
Optimize Threshold (External)			
Threshold value	0.363	0.695	0.390
Sensitivity	0.571	0.548	0.571
Specificity	0.931	0.888	0.925
PPV	0.400	0.284	0.381
NPV	0.964	0.960	0.964
Youden J	0.502	0.436	0.496
Generalizability Performance			
Training AUC	0.757	0.842	0.820
CV AUC	0.749	0.750	0.757
External AUC	0.757	0.742	0.744
Overfitting (Train–CV)	+0.008	+0.092	+0.063
Generalization Gap (CV–Ext)	–0.008	+0.008	+0.013

ROC–AUC, receiver operating characteristic–area under the curve; CI, confidence interval; PPV, positive predictive value; NPV, negative predictive value. The fixed threshold denotes the application of the optimal thresholds determined using the Youden J index in the 2010 training set to the 2017 external set. The optimized threshold represents the thresholds redefined using the Youden J index in the 2017 external set. Owing to the low prevalence of the external set (7.5%; 16.0% in the training set), the PPV values are low as expected. Overfitting: training AUC − CV AUC (a positive value indicates overfitting of the model to the training set). Generalization gap: CV AUC − External AUC (a positive value indicates performance loss in the external set, whereas a negative value indicates relatively better performance in the external set). DeLong test results: LR versus RF (Z = 0.539, *p* = 0.590), LR versus XGB (Z = 0.502, *p* = 0.616), and RF versus XGB (Z = −0.123, *p* = 0.902).

## Data Availability

The data that support the findings of this study are available from Ministry of Health of the Republic of Turkey but restrictions apply to the availability of these data, which were used under license for the current study, and so are not publicly available. Data are however available from the authors upon reasonable request and with permission of Ministry of Health of the Republic of Türkiye.

## Supplementary Materials

The following supporting information can be downloaded at: https://www.mdpi.com/article/10.3390/nu18142380/s1, Supplementary Table S1. Composite Cardiometabolic Risk Score: Definition, components, and rationale; Supplementary Table S2. Post-cleaning IQR method for outlier analysis and multivariate outlier analysis with Mahalanobis distance; Supplementary Table S3. Diagnostic performance of anthropometric indices in cardiometabolic risk prediction; Supplementary Table S4. Initial logistic regression model and performance; Supplementary Table S5. Calculation of sensitivity, specificity, positive, and negative predictive values; Supplementary Table S6. Initial logistic regression confusion matrix; Supplementary Table S7. Random forest model comparison and variable importance ranking according to sampling methods; Supplementary Table S8. XGBoost model comparison and variable importance; Supplementary Table S9. Machine learning model performance comparison (2010 training set, *n* = 1357); Supplementary Table S10. Internal and external validity performance of the models; Supplementary Table S11. Sensitivity analyses for predictor–outcome overlap (incorporation bias): single-predictor discrimination by outcome definition, and final logistic model with versus without VAI; Supplementary Table S12. Software and R package versions used for analysis; Supplementary Table S13. TRIPOD checklist for prediction model development and validation; Supplementary Figure S1. Calibration of the final logistic regression model in the development and external validation cohorts; Supplementary Figure S2. Decision curve analysis of the final logistic regression model in the development and external validation cohorts.

## Abbreviations

The following abbreviations are used in this manuscript:

TNHS	Türkiye Nutrition and Health Survey
CMR	Cardiometabolic Risk
BMI	Body Mass Index
TMI	Triponderal Mass Index
VAI	Visceral Adiposity Index
LAP	Lipid Accumulation Product
HDL-C	High-Density Lipoprotein Cholesterol
LDL-C	Low-Density Lipoprotein Cholesterol
SD	Standard Deviation
