# Supplementary material for: Estimation of Cardiometabolic Risk in Turkish Adolescents Using Different Anthropometric Techniques: Development and Temporal Validation of a Machine Learning Model
Source: Nutrients. 2026 Jul 21;18(14):2380. doi: 10.3390/nu18142380 (PMC13415240; doi:10.3390/nu18142380)
Supplement: Supplementary file 1 [file nutrients-18-02380-s001.zip › nutrients-4423967-supplementary.pdf]

Supplementary Table S1. Composite Cardiometabolic Risk Score: Definition, components, and rationale

Parameter	Reference Range	Anomaly Definition	n (%)	OR	95% CI	φ Correlations *(G/ TC/ LDL/ HDL/ TG)*
Glucose	70–110 mg/dL	<70 or >110	183 (13.5)	10.22	7.23–14.46	1 / 0.03 / −0.04 / 0.11 / −0.01
Total Cholesterol	<200 mg/dL	≥200	43 (3.2)	∞*	–	0.03 / 1 / 0.30 / −0.06 / 0.14
LDL Cholesterol	<100 mg/dL	≥100	360 (26.5)	6.70	4.91–9.14	−0.04 / 0.30 / 1 / −0.06 / 0.05
HDL Cholesterol	35–85 mg/dL	<35 or >85	303 (22.3)	12.52	9.01–17.39	0.11 / −0.06 / −0.06 / 1 / 0.18
Triglycerides	<150 mg/dL	≥150	61 (4.5)	30.73	15.68–60.21	−0.01 / 0.14 / 0.05 / 0.18 / 1

Anomaly Number	n (%)	Threshold Interpretation
0 parameter	661 (48.7)	—
1 parameter	479 (35.3)	—
**≥2** parameter**s**	**217 (16.0)**	**Prespecified threshold**
≥3 parameters	33 (2.4)	*Higher-burden category*

Combination	n (%)
Everything is normal	661 (48.7)
Only LDL	225 (16.6)
Only HDL	158 (11.6)
Only Glucose	85 (6.3)
Glucose + HDL	54 (4.0)
LDL + HDL	49 (3.6)

CMR is defined as at least two of five biochemical parameters being outside the reference range. OR: Odds ratio of the abnormality of each parameter to CMR; φ: Phi correlation coefficients between parameters; Combination codes: G=Glucose, T=Total Cholesterol, L=LDL, H=HDL, R=Triglycerides abnormal, -=normal. *: All individuals with total cholesterol abnormality (n=43) were included in the CMR positive group. The ≥2 threshold was defined a priori based on the concept of metabolic clustering and prior literature. The resulting prevalence (16.0%) and number of cases (n = 217) are reported descriptively and were not used to optimize the threshold.

Supplementary Table S2. Post-cleaning IQR method for outlier analysis and multivariate outlier analysis with Mahalanobis distance

Variable	TNHS 2010 (n)	Outlier Value (n)	Outlier Value (%)	TNHS 2017 (n)	Outlier Value (n)	Outlier Value (%)
Body weight	1627	25	1.54	689	17	2.47
Height/length	1630	7	0.43	689	2	0.29
Waist circumference	1625	56	3.45	679	21	1.77
Hip circumference	1624	20	1.23	677	20	2.95
BMI	1621	48	2.96	688	18	2.62
WHO BMI Z-score	1545	15	0.97	683	4	0.59
Waist/hip	1631	35	2.15	677	9	1.33
Waist/height	1625	67	4.12	679	15	2.21
Glucose	1467	46	3.14	618	23	3.72
Total cholesterol	1473	27	1.83	601	8	1.33
LDL cholesterol	1466	19	1.30	609	10	1.64
HDL cholesterol	1475	37	2.51	615	12	1.95
Triglycerides	1471	67	4.55	617	30	4.86

Data Set	Sex	Analyzed	Outlier Value (n)	Outlier Value (%)
TNHS 2010	Men	786	35	4.45
	Women	814	39	4.79
TNHS 2017	Men	329	12	3.65
	Women	328	9	2.74

Critical value (p<0.001): 24.32

Supplementary Table S3. Diagnostic performance of anthropometric indices in cardiometabolic risk prediction

Predictor	AUC	95% CI	Cutoff Point	Sensitivity	Specificity	PPV	NPV	Youden J	LR+	LR−
WHO BMI z-score	0.615	0.571–0.658	0.545	49.3%	70.5%	24.2%	88.0%	0.198	1.67	0.72
Waist-to-height ratio	0.618	0.575–0.661	0.465	48.8%	72.9%	25.5%	88.2%	0.217	1.80	0.70
Waist-to-hip ratio	0.542	0.500–0.585	0.818	45.2%	62.4%	18.6%	85.7%	0.075	1.20	0.88
Triponderal mass index	0.620	0.578–0.663	13.468	47.9%	72.1%	24.6%	87.9%	0.200	1.72	0.72
Lipid accumulation product index	0.670	0.626–0.714	13.020	47.0%	82.2%	33.4%	89.1%	0.292	2.64	0.64
Visceral adiposity index	0.747	0.707–0.787	1.635	55.3%	87.0%	44.8%	91.1%	0.423	4.26	0.51

n = 1357. AUC, area under the curve; CI, confidence interval; PPV, positive predictive value; NPV, negative predictive value; LR+, positive likelihood ratio; LR−, negative likelihood ratio; J, Youden index. AUC comparisons are validated using the DeLong test, and confidence intervals are validated using bootstrap (n = 2,000).

Supplementary Table S4. Initial logistic regression model and performance

Predictor	β	SE	OR	95% CI	p
**Constant**	−2.481	1.057	0.08	0.01–0.72	0.019
WHO BMI z-score	0.100	0.103	1.1	0.91–1.39	0.334
Waist-to-height ratio	−1.331	3.350	0.26	0.00–184.89	0.691
Waist-to-hip ratio	−0.361	1.493	0.70	0.04–12.93	0.809
Triponderal mass index	0.004	0.026	1.00	0.90–1.04	0.886
LAP index	0.005	0.010	1.01	0.99–1.03	0.620
**VAI**	**1.132**	**0.125**	**3.10**	**2.44–3.99**	**<0.001**

**General Performance**		**Optimal Threshold Performance**	
**10-Fold CV AUC**:	0.734	**Threshold value**	0.211
**Training AUC (95%)**:	0.746 (0.706–0.787)	**Sensitivity**	0.544
**PR-AUC**:	0.503	**Specificity**	0.876
**Brier Score**:	0.107	**PPV/NPV**	0.456/0.910

Threshold	Sensitivity	Specificity	PPV	NPV	Youden J
0.10	0.770	0.546	0.244	0.926	0.315
0.15	0.613	0.772	0.338	0.913	0.385
**0.20***	**0.548**	**0.863**	**0.433**	**0.909**	**0.412**
0.25	0.470	0.910	0.498	0.900	0.380
0.30	0.410	0.938	0.556	0.893	0.348
0.50	0.235	0.981	0.699	0.871	0.216

Predictor	Significance (%)
**1. VAI**	**100.0**
2. WHO BMI z-score	9.3
3. LAP index	4.0
4. Waist-to-height ratio	2.9
5. Waist-to-hip ratio	1.1
6. Triponderal mass index	0.0

n = 1,357. Dependent variable: composite cardiometabolic risk (≥2 abnormal biochemical parameters). Model fit statistics: Nagelkerke $R^2 = 0.245$, Hosmer–Lemeshow $\chi^2 = 11.13$ ($p = 0.195$), likelihood ratio $\chi^2 = 210.23$ ($p < 0.001$), AIC = 996.64. OR, odds ratio; CI, confidence interval. Bold values indicate statistically significant results ($p < 0.05$). CV, cross-validation; PR-AUC, area under the precision-recall curve; PPV, positive predictive value; NPV, negative predictive value. *: Optimal threshold (Youden J maximum)

Supplementary Table S5. Calculation of sensitivity, specificity, positive, and negative predictive values

Value	Definition	Calculation
Sensitivity (TP/[TP + FN])	The question is how many of the true positives are actually detected.	$\frac{118}{118+99} = 0.5438$
Specificity (TN/[TN + FP])	The question is how many of the true negatives have been correctly excluded.	$\frac{999}{999+141} = 0.8763$
Positive predictive value (TP/[TP + FP])	It is the probability that a positive test result is actually positive.	$\frac{118}{118+141} = 0.4556$
Negative predictive value (TN/[TN + FN])	It is the probability that a negative test result is actually negative.	$\frac{999}{999+99} = 0.9098$

Supplementary Table S6. Initial logistic regression confusion matrix

	**True Values**		
**Test Result**	**True Positive (CMR+)**	**True Negative (CMR–)**	**Total**
**Risky (+)**	118 *(True Positive)*	141 *(False Positive)*	259
**Not risky (–)**	99 *(False Negative)*	999 *(True Negative)*	1098
**Total**	217	1140	1357

Optimal threshold value: 0.211

Supplementary Table S7. Random forest model comparison and variable importance ranking according to sampling methods

**Model**	**Optimal Parameters**	**CV AUC**	**CV Sensitivity**	**CV Specificity**	**CV Youden J**
RF default*	mtry = 3, min.node.size = 10 *(splitrule = gini)*	0.715	0.272	0.969	0.241
RF downsampling	mtry = 3, min.node.size = 30	**0.728**	0.788	0.585	0.373
RF upsampling	mtry = 1, min.node.size = 30	0.725	**0.896**	0.465	0.361
RF SMOTE	mtry = 3, min.node.size = 30	0.724	0.840	0.532	0.371
Logistic Regression	-	**0.734**	0.544	**0.876**	**0.420**
**Order**	**Predictor**	**Downsampling**	**Upsampling**	**SMOTE**	**Mean**
1	VAI	100.0	100.0	100.0	**100.0**
2	LAP index	30.3	41.3	37.6	**36.4**
3	WHO BMI z-score	6.6	9.5	5.4	7.1
4	Waist-to-height ratio	8.8	9.2	3.3	7.1
5	Triponderal mass index	6.0	2.9	4.4	4.4
6	Waist-to-hip ratio	0.0	0.0	0.0	0.0

Supplementary Table S8. XGBoost model comparison and variable importance

Model	Optimal Parameters	CV AUC	CV Sensitivity	CV Specificity	CV Youden J
XGB default *	nrounds = 100, max_depth = 6, eta = 0.3, gamma = 0, min_child_weight = 1.	0.693	0.951	0.273	0.223
XGB downsampling	nrounds = 50, max_depth = 3, eta = 0.05, gamma = 0, min_child_weight = 5.	0.728	0.799	0.576	0.375
XGB upsampling	nrounds = 50, max_depth = 4, eta = 0.1, gamma = 1, min_child_weight = 10.	0.735	0.836	0.516	0.352
XGB SMOTE	nrounds = 50, max_depth = 2, eta = 0.1, gamma = 0, min_child_weight = 10	0.734	0.825	0.549	0.374
Logistic Regression	-	**0.734**	0.544	**0.876**	**0.420**

Order	Predictor	Downsampling	Upsampling	SMOTE	Mean
1	VAI	100.0	100.0	100.0	100.0
2	LAP index	7.5	30.0	31.4	23.0
3	WHO BMI z-score	4.5	6.7	4.3	5.0
4	Waist-to-height ratio	2.7	6.6	5.5	3.3
5	Triponderal mass index	0.4	4.0	2.8	2.9
6	Waist-to-hip ratio	0.0	0.0	0.0	0.0

Supplementary Table S9. Machine learning model performance comparison (2010 training set, n = 1357)

Metric	Logistic Regression	Random Forest	XGBoost	XGBoost (Regularized)	Winning Model
**Discrimination**					
ROC–AUC	0.757	0.842	**0.879**	**0.820**	XGBoost
PR-AUC	0.508	0.565	**0.627**	**0.557**	XGBoost
**Classification**					
Optimal threshold	0.161	0.439	0.242	**0.164**	—
Sensitivity	0.627	**0.820**	0.783	**0.673**	Random forest
Specificity	0.783	0.705	**0.808**	**0.832**	XGBoost (Reg.)
PPV	0.354	0.346	**0.437**	**0.432**	XGBoost
NPV	0.917	**0.954**	0.951	**0.930**	Random forest
**General Performance**					
Accuracy	0.758	0.724	**0.804**	**0.806**	XGBoost (Reg.)
Balanced accuracy	0.705	0.763	**0.796**	**0.752**	XGBoost
F1-score	0.453	0.487	**0.561**	**0.440**	XGBoost
Youden J	0.409	0.526	**0.591**	**0.504**	XGBoost
MCC	0.333	0.397	**0.479**	**0.412**	XGBoost
**Calibration**					
Brier score	0.107	0.176	**0.102**	**0.110**	XGBoost
**Clinical Benefit**					
LR+	2.88	2.78	**4.08**	**4.00**	XGBoost
LR−	0.48	**0.25**	0.27	**0.35**	Random forest
**Model**	**Logistic Regression**	**Random Forest**	**XGBoost**		
CV AUC	0.749	0.750	**0.769**	**0.757**	—
Training AUC	0.757	0.842	**0.879**	**0.820**	—
**Overfitting**					
Difference	+0.008	+0.092	**+0.110**	**+0.062**	—
Interpretation	Stable	Slightly overfit	Overfit	Controlled (well regulated)	—
**Variable Importance**					
VAI	**100.0**	**100.0**	**69.4**	**68.0**	—
LAP	4.0	29.7	13.7	**15.0**	—
WHO BMI-z	9.3	0.0	7.5	**8.0**	—
Waist-to-height ratio	2.9	7.7	6.6	**6.0**	—
Age (standardized)	12.8	0.2	2.9	**3.0**	—

Supplementary Table S10. Internal and external validity performance of the models

Model	CV AUC	External AUC	Difference	DeLong p (According to LR)
Logistic regression	0.749	0.757	−0.008	—
Random forest	0.750	0.742	+0.008	0.590
XGBoost (Reg.)	0.757	0.744	+0.013	0.616

Supplementary Table S11. Sensitivity analyses for predictor–outcome overlap (incorporation bias)

**Panel A. Single-predictor discrimination (AUC, 95% CI) by outcome definition (TNHS 2010, n = 1,357)**			
Predictor	***Original (≥2 of 5)***	***Non-lipid (≥1 of 3)***	***Non-lipid (≥2 of 3)***
VAI	*0.747 (0.707–0.787)*	*0.572 (0.541–0.603)*	*0.620 (0.545–0.695)*
LAP	*0.670 (0.626–0.714)*	*0.564 (0.531–0.597)*	*0.636 (0.562–0.710)*
WHO BMI z-score	*0.615 (0.571–0.658)*	*0.567 (0.535–0.600)*	*0.588 (0.514–0.661)*
Waist-to-height ratio	*0.618 (0.575–0.661)*	*0.592 (0.560–0.624)*	*0.596 (0.523–0.669)*
Triponderal mass index	*0.620 (0.578–0.663)*	*0.590 (0.558–0.621)*	*0.604 (0.532–0.676)*
Waist-to-hip ratio	*0.542 (0.500–0.585)*	*0.515 (0.483–0.547)*	*0.496 (0.424–0.569)*

**Panel B. Final logistic regression model with versus without VAI**				
Set	***AUC (full)***	***AUC (without VAI)***	***ΔAUC***	***DeLong p***
Development (2010)	*0.757*	*0.658*	*0.099*	*<0.001*
External (2017)	*0.757*	*0.683*	*0.074*	*0.10*

Number of positive cases: original outcome n = 217; non-lipid ≥1 n = 503; non-lipid ≥2 n = 75. The non-lipid outcome uses glucose, total cholesterol, and LDL-C only, which share no components with VAI or LAP. AUC, area under the ROC curve; CI, confidence interval; VAI, visceral adiposity index; LAP, lipid accumulation product.

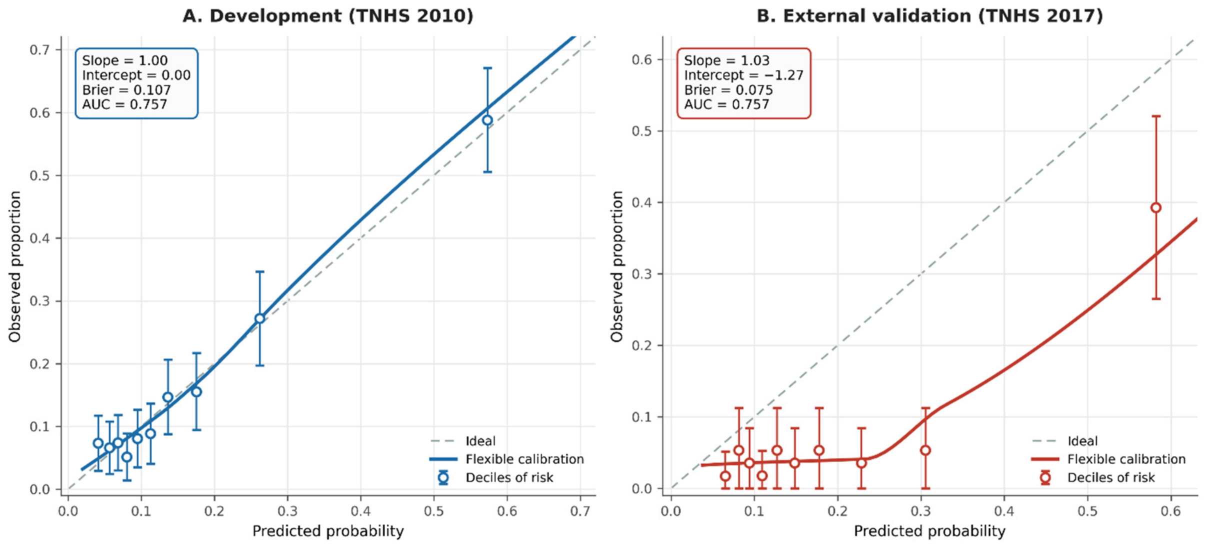

Supplementary Figure S1. Calibration of the final logistic regression model. (A) Development cohort (TNHS 2010, n = 1,357) and (B) external temporal validation cohort (TNHS 2017, n = 561). Open circles denote observed event proportions across deciles of predicted risk (with 95% confidence intervals); the solid line is the flexible (loess) calibration curve; the dashed diagonal represents ideal calibration. In the development cohort, calibration was near-perfect (slope = 1.00, intercept = 0.00). In the external cohort, the calibration slope remained close to 1 (1.03), indicating preserved predictor effects, whereas the negative calibration intercept (−1.28) reflects systematic over-prediction attributable to the lower outcome prevalence in 2017 (7.5% vs 16.0%); this calibration-in-the-large offset can be corrected by simple intercept recalibration without refitting the model.

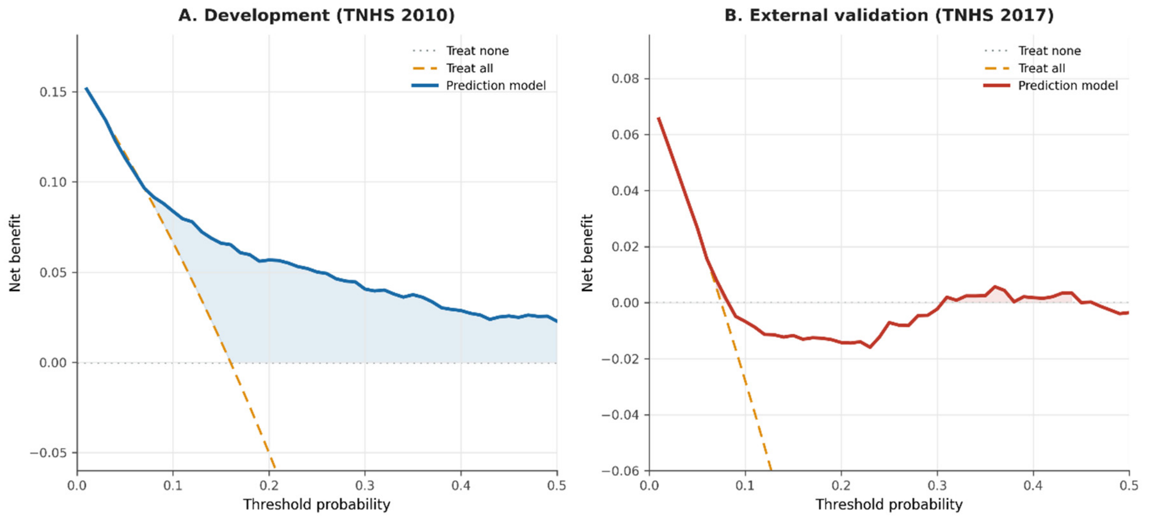

Supplementary Figure S2. Decision curve analysis of the final logistic regression model. (A) Development cohort (TNHS 2010) and (B) external temporal validation cohort (TNHS 2017). The solid line shows the net benefit of the prediction model across threshold probabilities; the dashed line represents the "treat all" strategy and the dotted line the "treat none" strategy. In the development cohort, the model provided a positive net benefit exceeding both reference strategies across the clinically relevant threshold range (approximately 0.10–0.30). In the external cohort, absolute net benefit was smaller owing to the lower outcome prevalence and the calibration offset noted in Supplementary Figure S1, with the model remaining at least as beneficial as the reference strategies at low thresholds.

Supplementary Table S12. Software and R package versions used for analysis

Software / Package	Version	Purpose in analysis
R	4.4.2	Statistical computing environment
tidyverse	2.0.0	Data management and processing
caret	7.0-1	Model training and cross-validation
tidymodels	1.4.1	Machine-learning workflow (XGBoost)
tune	2.0.1	Hyperparameter tuning
finetune	1.2.1	Bayesian hyperparameter optimization
ranger	0.17.0	Random forest implementation
randomForest	4.7-1.2	Random forest (supporting)
xgboost	1.7.8.1	Gradient boosting model
themis	1.0.3	Class-imbalance correction (SMOTE)
smotefamily	1.4.0	Class-imbalance correction
pROC	1.18.5	ROC curve analysis and DeLong test
PRROC	1.4	Precision–recall curve analysis
pmsampsize	1.1.3	Sample size for prediction model development
ResourceSelection	0.3-6	Hosmer–Lemeshow calibration test
car	3.1-3	Multicollinearity (VIF) assessment
SHAPforxgboost	0.1.3	SHAP value computation
shapviz	0.10.3	SHAP visualization
ggplot2	4.0.0	Data visualization
patchwork	1.3.0	Figure composition
readxl	1.4.5	Data import
openxlsx	4.2.7.1	Data export

Analyses were performed in R version 4.4.2 (2024-10-31) on Windows 11 x64. Only the principal packages used in the analysis are listed; a complete session record (including all dependencies) is available from the authors on request.

Supplementary Table S13. TRIPOD Checklist: Prediction Model Development and Validation

Section / Topic	Item	Checklist Item	Reported (Section)
**Title and Abstract**			
Title	1	Identify the study as developing and/or validating a multivariable prediction model, the target population, and the outcome.	Title
Abstract	2	Provide a summary of objectives, study design, setting, participants, sample size, predictors, outcome, statistical analysis, results, and conclusions.	Abstract
**Introduction**			
Background	3a	Explain the medical context and rationale for developing or validating the model, including references to existing models.	Introduction
Objectives	3b	Specify the objectives, including whether the study describes development, validation, or both.	Introduction (final paragraph)
**Methods**			
Source of data	4a	Describe the study design or source of data, separately for development and validation sets.	Methods, Study Design and Population
Source of data	4b	Specify key study dates.	Methods (TNHS 2010 and 2017)
Participants	5a	Specify key elements of the study setting, including number and location of centres.	Methods, Study Design and Population
Participants	5b	Describe eligibility criteria for participants.	Methods; Figure 1
Participants	5c	Give details of treatments received, if relevant.	Not applicable (observational survey)
Outcome	6a	Clearly define the outcome predicted, including how and when assessed.	Methods, Data Analysis (composite CMR, ≥2 of 5)
Outcome	6b	Report any actions to blind assessment of the outcome.	Methods (outcome defined by objective biochemical thresholds)
Predictors	7a	Clearly define all predictors, including how and when measured.	Methods, Anthropometric Measurements and Biochemical Findings
Predictors	7b	Report any actions to blind assessment of predictors.	Methods (predictors measured independently of outcome)
Sample size	8	Explain how the study size was arrived at.	Methods, Data Analysis (pmsampsize); Supplementary Table S12
Missing data	9	Describe how missing data were handled.	Methods, Study Design and Population (complete-case); Figure 1
Statistical analysis	10a	Describe how predictors were handled in the analyses.	Methods, Data Analysis
Statistical analysis	10b	Specify type of model, all model-building procedures, and internal validation method.	Methods, Data Analysis (LR, RF, XGBoost; repeated 10-fold CV; tuning)
Statistical analysis	10c	For validation, describe how predictions were calculated.	Methods, Data Analysis (2010 model applied to 2017)
Statistical analysis	10d	Specify all measures used to assess model performance.	Methods, Data Analysis (AUC, calibration slope/intercept, Brier, DCA)
Statistical analysis	10e	Describe any model updating (e.g., recalibration) arising from validation.	Discussion; Supplementary Figure S1 (recalibration recommended)
Risk groups	11	Provide details on how risk groups were created, if done.	Results and Discussion (screening vs confirmatory thresholds)
Development vs validation	12	For validation, identify differences from development data in setting, eligibility, outcome, predictors.	Methods; Discussion (age range, prevalence differences)
**Results**			

Participants	13a	Describe the flow of participants, including numbers with and without the outcome.	Results; Figure 1
Participants	13b	Describe the characteristics of the participants, including numbers with missing data.	Results, Table 1; Figure 1
Participants	13c	For validation, compare distribution of important variables with development data.	Results, Table 1; Discussion
Model development	14a	Specify the number of participants and outcome events in each analysis.	Results; Methods (n = 1,357; 217 events)
Model development	14b	Report the unadjusted association between each candidate predictor and outcome, if done.	Results; Figure 3; Supplementary Table S3
Model specification	15a	Present the full prediction model (all coefficients and intercept).	Results (logistic regression equation); Table 2
Model specification	15b	Explain how to use the prediction model.	Results and Discussion (simplified formula for calculator)
Model performance	16	Report performance measures (with CIs) for the model.	Results, Table 3; Supplementary Figures S1, S2
Model updating	17	If done, report results from any model updating.	Not applicable (updating recommended, not performed)
**Discussion**			
Limitations	18	Discuss any limitations of the study.	Discussion, Limitations
Interpretation	19a	For validation, discuss results with reference to development performance.	Discussion
Interpretation	19b	Give an overall interpretation of the results.	Discussion
Implications	20	Discuss the potential clinical use and implications for future research.	Discussion; Conclusions
**Other Information**			
Supplementary information	21	Provide information about availability of supplementary resources.	Supplementary Materials
Funding	22	Give the source of funding and role of funders.	Funding statement

Checklist based on Collins GS, Reitsma JB, Altman DG, Moons KGM. Transparent Reporting of a multivariable prediction model for Individual Prognosis Or Diagnosis (TRIPOD). Ann Intern Med. 2015;162:55–63. D, development; V, validation.
